# Taste-Odor Association Learning Alters the Dynamics of Intraoral Odor Responses in the Posterior Piriform Cortex of Awake Rats

**DOI:** 10.1523/ENEURO.0010-23.2023

**Published:** 2023-03-24

**Authors:** Joost X. Maier, Ammar Idris, Brooke A. Christensen

**Affiliations:** Department of Neurobiology and Anatomy, Wake Forest Atrium Baptist Medical Center, Winston-Salem, NC 27157

**Keywords:** olfactory cortex, taste, smell, flavor, retronasal, decoding

## Abstract

How an odor is perceived is to a large extent dependent on the context in which that odor is (or has been) experienced. For example, experiencing an odor in mixture with taste during consumption can instill taste qualities in the percept of that odor (e.g., vanilla, an odor, has a gustatory quality: sweet). How associative features of odors are encoded in the brain remains unknown, but previous work suggests an important role for ongoing interactions between piriform cortex and extraolfactory systems. Here, we tested the hypothesis that piriform cortex dynamically encodes taste associations of odors. Rats were trained to associate one of two odors with saccharin; the other odor remained neutral. Before and after training, we tested preferences for the saccharin-associated odor versus the neutral odor, and recorded spiking responses from ensembles of neurons in posterior piriform cortex (pPC) to intraoral delivery of small drops of the same odor solutions. The results show that animals successfully learned taste-odor associations. At the neural level, single pPC neuron responses to the saccharin-paired odor were selectively altered following conditioning. Altered response patterns appeared after 1 s following stimulus delivery, and successfully discriminated between the two odors. However, firing rate patterns in the late epoch appeared different from firing rates early in the early epoch (<1 s following stimulus delivery). That is, in different response epoch, neurons used different codes to represent the difference between the two odors. The same dynamic coding scheme was observed at the ensemble level.

## Significance Statement

Odors carry important meaning beyond their chemical identity. One particularly salient example of this are food odors, which play an important role in determining flavor preferences and food choice behavior. How these extraolfactory aspects of odor are represented is unknown. Using extracellular recordings in awake rats in the context of a flavor preference learning task, we show that learned taste associations of odor stimuli are represented in the dynamic firing patterns of posterior piriform cortex (pPC) neurons. The results suggest that associative odor coding results from ongoing interactions between olfactory and extraolfactory systems.

## Introduction

Smell is unique among the senses in that it faces an extraordinary large number of potential sensory qualities (Bushdid et al., 2014). To meet this challenge, olfactory space is not represented in fixed, topographically organized maps as in other sensory systems (Rennaker et al., 2007; Stettler and Axel, 2009). Instead, cortical odor coding is highly plastic and depends to a large extent on the context in which an individual experiences an odor (Wilson and Stevenson, 2003; Wilson et al., 2004).

One particularly salient context in which olfaction plays a major role is consumption. During consumption, odor stimuli are typically experienced in mixture with taste. Behavioral work has shown that interactions between taste and odor components of flavor are highly adaptive in informing consumption decisions (Holman, 1975; Sclafani and Ackroff, 1994; Slotnick et al., 1997; Blankenship et al., 2019; Elliott and Maier, 2020; Maier and Elliott, 2020). One example of taste-odor interactions during consumption is the formation of taste-odor associations. Odors experienced in mixture with taste acquire qualities associated with that taste (Stevenson et al., 1995, 1998, 2000a, b; Stevenson and Boakes, 2004; Yeomans et al., 2006; McQueen et al., 2020). Taste-odor association learning is robust, rapid, and affects subsequent consumption behavior: odors that have been experienced in mixture with palatable tastes become attractive.

Recent work aimed at elucidating how taste-odor associations are represented in the brain has indicated a role for the insular gustatory cortex (GC). Optogenetic inhibition of GC after rats learned to associate an odor with saccharin (a sweet taste) impaired their ability to express a preference for that odor (Maier et al., 2015; Blankenship et al., 2019). However, how GC exerts its effect on neural coding of taste-odor associations is unknown. Taste-odor associations may reside locally in GC. Alternatively, GC may support taste-odor associations by modulating sensory processing in olfactory areas. One potential area through which GC may affect olfactory processing is the piriform (olfactory) cortex, a large cortical surface that receives bottom-up input from the olfactory bulb representing a vast space of odorant molecules (Scott et al., 1980; Schwob and Price, 1984; Rennaker et al., 2007; Stettler and Axel, 2009; Miyamichi et al., 2011; Sosulski et al., 2011). Moreover, the piriform cortex is ideally situated to interact with extraolfactory systems (Luskin and Price, 1983; Johnson et al., 2000; Haberly, 2001; Majak et al., 2004; Sadrian and Wilson, 2015), sculpting odor representations based on the context an odor is encountered in (Kadohisa and Wilson, 2006; W. Li et al., 2006, 2008; Calu et al., 2007; Roesch et al., 2007; Barnes et al., 2011; Chen et al., 2011; Chapuis and Wilson, 2012; Gire et al., 2013; Meissner-Bernard et al., 2019; D. Wang et al., 2019). Interactions with extraolfactory systems are particularly pronounced in the posterior piriform cortex (pPC; Johnson et al., 2000; Majak et al., 2004; Zelano et al., 2005; W. Li et al., 2006; Calu et al., 2007). Indeed, pPC odor representations have been shown to be affected by experience and multisensory context (Karunanayaka et al., 2015; Avery et al., 2020). Previous work from our lab identified GC as a source of extraolfactory input to pPC, as inactivation of GC affects pPC responses to taste stimuli, as well as odor stimuli even in the absence of concurrent taste input (Maier et al., 2015). Together, these findings suggest that encoding of taste-odor associations may be supported by ongoing interactions between GC and pPC.

Here, we test the hypothesis that taste associations of odor stimuli are represented in pPC. Specifically, we predict that experience with taste-smell mixtures affects responses of pPC neurons to intraoral delivery of odor solutions. We further predict that the effect of experience is reflected in changes in response dynamics, consistent with the idea that odor coding in the context of consumption depends on ongoing interactions with the taste system. Alternatively, if taste associations are represented locally in GC, we do not predict any experience-dependent changes in responsiveness at the level of pPC. Associations may also be represented locally in pPC, independently of GC. In this case, we expect experience to be reflected in static pPC response patterns. To test our predictions regarding neural coding of taste-odor associations, we used extracellular electrophysiology to record spiking activity from ensembles of single pPC neurons of awake rats while they consumed odor solutions, before and after taste-odor association learning. We show that taste-odor association learning changes the temporal dynamics of odor responses, such that initial odor-selective response patterns are transformed over the course of seconds into a novel representation that distinguishes taste-associated from taste-naive odors. These results suggest that pPC dynamically encodes both odor identity and taste association.

## Materials and Methods

### Subjects

A total of 30 adult female Long–Evans rats (Charles River), weighing between 250 and 300 g at the time of surgery served as subjects. All rats were individually housed and kept on a 12/12 h light/dark cycle [zeitgeber time 0 = 6 A.M.]. Experiments were conducted during the light cycle. All animal procedures were performed in accordance with the Wake Forest Atrium Baptist Medical Center animal care committee’s regulations.

### Surgery

Animals were injected intraperitoneally with the analgesic meloxicam (10 mg/kg) before surgery. Stereotaxic surgery was performed under intranasally administered isoflurane anesthesia (2–5%). First, the scalp was treated with lidocaine ointment, an incision was made, and the skin retracted from the skull. Using a dental drill, a craniotomy was made overlying the posterior piriform olfactory cortex (unilateral or bilateral): 1.4 mm posterior to bregma, 5.2–5.6 mm lateral to the midline, 6.4–7.4 mm ventral from the surface of the brain (Paxinos and Watson, 1986). Five additional burr holes were made, evenly spread across the skull, for the insertion of skull screws that provide stability to the implant. Electrodes were then lowered to the piriform cortex over the course of 30 min using a manually-driven stereotaxic arm. Once in place, the craniotomy was filled with Kwik-Cast (World Precision Instruments), and the electrode connector and skull screws were covered in dental acrylic, fixing the electrodes in place. Electrode assemblies consisted of micro-wire arrays with 6 or 8 electrodes in circular arrangement, or 16 electrodes in square arrangement. Electrodes were 25-μm diameter stainless steel wires, spaced 100–200 μm apart (MicroProbes), or multielectrode silicon probes (two shanks of 16 electrode contacts each, shanks spaced 500 μm apart; electrode contacts spaced 50 μm apart, NeuroNexus model A2x16-10 mm-50-500-177-CM32). Intraoral cannulae (IOC) were implanted to provide access to the oral cavity (Phillips and Norgren, 1970): flanged microbore PE tubing (1.143 mm ID, 1.574 mm OD) was inserted behind the second molar using a 20-G needle, and guided under the skin overlying the zygomatic arch to exit at the edge of the cranial implant. Tubing was then attached to a coupler body (CPC, #SMF01) that could interface with a counterpart holding a manifold of fluid delivery tubes. Once in place, the coupler was secured to the rest of the implant with dental acrylic. Animals recovered in their home cage with *ad libitum* access to water and mashed rat chow for 4–5 d after surgery before the start of the experiment.

### Stimuli

Unisensory odor stimuli were exemplars of monomolecular odorants (obtained from Sigma-Aldrich; >98% purity) that have been used in similar behavioral (Blankenship et al., 2019) and neural recording (Maier, 2017) paradigms in previous studies: methyl valerate and 2-hexanone in aqueous solution (0.025% volume/volume in distilled water). Saccharin (0.2%; Fisher Scientific) was used as an unconditioned stimulus during conditioning sessions. All stimuli were presented intraorally to allow, as much as possible, natural sensory stimulation dynamics associated with intraoral evaluation of flavor. There is no empirical evidence that the odorants used here completely lack gustatory and/or trigeminal qualities. However, similar concentrations of odor solutions have been shown to be below nonolfactory behavioral detection thresholds (Slotnick et al., 1997; Gautam and Verhagen, 2012) and are therefore unlikely to have contributed to the observed responses.

### Behavioral procedures

#### Preference testing

Two-bottle tests were used to assess odor preferences before and after conditioning. During each preference testing session, animals were given free access to two odor solutions overnight (6 P.M. to 8 A.M.). Animals received *ad libitum* access to water in between two-bottle tests, ensuring that they were never deprived of fluid during preference testing sessions. Relative position of the odor bottles in the cage was alternated between sessions and counterbalanced across rats. In one preference test, consumption from one bottle could not be determined because of spillage (0.8% of all bottles); data from this animal were excluded from contributing to the behavioral dataset.

#### Conditioning

One-bottle access was used for conditioning, performed over four consecutive days. Each morning (10 A.M.), animals had access to 10 ml of one of the odors (odor A) in plain water, or the other odor (odor B) in saccharin solution for 30 min. Bottles alternated on an A-B-A-B schedule, and the identity of odors A and B (methyl valerate or 2-hexanone) was counterbalanced across rats.

### Stimulus presentation and recording procedures

The recording arena consisted of a 29 × 23 × 33 cm Plexiglas chamber encased in metal that served as a Faraday cage. Stimuli were delivered via syringe pumps directly onto the dorsal surface of the tongue while animals were moving freely around the arena. Syringes containing stimulus solution were connected to blunted needles fitted with strands of PE tubing (1.143 mm ID, 1.574 mm OD) that fed into the recording arena via an opening in the roof. At the distal end of each strand of PE tubing, a 5 cm strand of PI microbore tubing (0.0254 mm ID, 0.0270 mm OD) was glued, and all tubes were inserted into the through hole of a coupler body (CPC, #SMF02) and held together with glue. Before experimental sessions, the collection of PI tubes was fed into the IOC and secured in place by mating the coupler bodies on the tube manifold and IOC. Once connected, the tips of the PI tubes extended 0.5 mm below the tip of the IOC into the oral cavity. Animals were habituated to the recording setup and stimulus delivery apparatus before recording by presenting drops of water through IOC in the recording arena. During recording sessions, airborne odorants were cleared by a continuously running fan mounted in the ceiling of the recording arena. To encourage consumption of stimuli, animals were deprived of water for 6 h before recording sessions. During each recording session, odors A and B, as well as plain water were presented in random order (10 repetitions of each stimulus). Stimuli were always presented in random order. Intraoral stimuli were delivered in 30- to 50-μl aliquots (total duration of delivery <100 ms), with a random intertrial interval ranging from 30–45 s, allowing sufficient time for animals to swallow the fluid and clear their mouth. Each session yielded between 1 and 16 (mean = 5.0) single neurons (0.3 neurons/electrode on average). In a subset of animals, multisensory mixtures of odors A/B and taste compounds (saccharin and/or sodium chloride), as well as unisensory taste compounds in isolation, were presented in addition to the stimuli listed above (data not included in the present paper). During these sessions, both odorants were paired with the same tastant(s) for 10 trials per mixture. Recording and preference testing sessions were always performed according to the same schedule. Given the limited and balanced nature of mixture exposure during recording sessions, it is unlikely to have impacted the results reported in the present study. Results from a subset of recordings in response to mixtures have been reported previously (Idris et al., 2023).

### Electrophysiological recording and data processing

The continuous extracellular signal recorded from each electrode was amplified, digitized and stored for offline analysis at a sampling rate of 25 kHz using INTAN RHD2000 hardware and acquisition software (Intan Technologies). Action potentials were extracted, clustered and sorted using the klusta/phy toolbox to obtain single neuron spike time stamps (Rossant et al., 2016). For silicon probe recordings, spikes were clustered taking into account the possibility that the same action potentials could be recorded by up to three neighboring channels. Spike time stamps were then binned at 1-ms resolution and aligned to stimulus delivery before further analysis. Only action potentials that exceeded 3.5 SD units of the high-pass filtered (400 Hz) voltage signal were included in the clustering analysis, and only clusters that contained <2% of action potentials occurring at an interspike interval of 2 ms or less were included in the dataset (Gadziola et al., 2015).

### Data analysis

Offline analyses were performed using MATLAB (MathWorks). For time-averaged analysis, responses were averaged over the following time windows relative to stimulus delivery: −2000–0 ms (baseline period), 0–2500 ms (total stimulus period), 0–1250 ms (early stimulus period), 1250–2500 ms (late stimulus period). For time-resolved analysis, responses were averaged in a sliding window over time (window size: 500 ms; step size: 50 ms). Cohen’s *d* was calculated to provide a continuous measure of effect size (discriminability) for each neuron: Cohen’s *d* = (mean_A_ − mean_B_)/std_pooled_, where std_pooled_ = ([std_A_^2^ + std_B_^2^])/2.

To make the connection between firing rates of individual neurons and the amount of information present in the collective responses of ensembles of neurons, we performed single-trial ensemble decoding analysis (Quiroga et al., 2007; Bolding and Franks, 2017). Specifically, we asked how well odors A and B could be discriminated based on the firing rates of all simultaneously-recorded single neurons during a given session. For each temporal analysis window (i.e., baseline, stimulus, sliding window over time), we first computed single-trial ensemble response vectors, consisting of the time-averaged response in that window for each simultaneously-recorded neuron. Each single-trial ensemble response vector was then compared with two templates (A and B), consisting of the ensemble response vectors averaged across all odor A and odor B trials (excluding the trial to be decoded). Similarity of each trial to both templates was then quantified by calculating the Euclidean distance. Decoding accuracy was defined as the percentage of trials that showed greater similarity with the correct template (i.e., an ensemble response on an odor A trial showing higher similarity with the odor A template than with the odor B template, and vice versa).

### Histology

**Figure 1.**
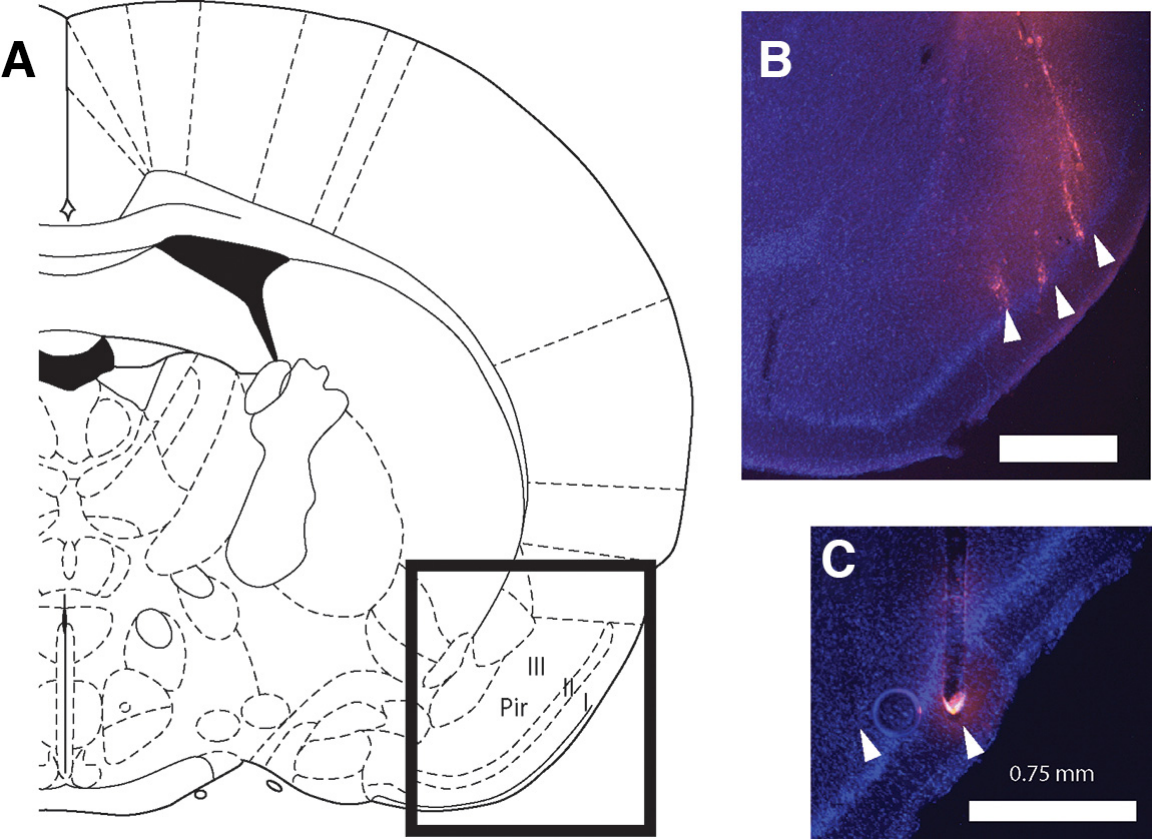
Histologic reconstruction of recording sites. ***A***, Schematic from a rat brain atlas (Paxinos and Watson, 1986) indicating the general region of the posterior piriform cortex Layers I, II, and III in a coronal view (1.4 mm posterior to bregma). ***B***, ***C***, Coronal sections taken from two rat brains showing DAPI (blue) and DiI (pink) staining of nuclei and electrode tracts, respectively. Scale bar indicates 0.75 mm. Electrode tips are indicated by arrowheads. Animal in ***B*** was implanted with a microwire array; animal in ***C*** with a silicon probe.

Electrodes were labeled with a drop of Vybrant DiI cell-labeling solution (Thermo Fisher Scientific), applied with a needle tip before implantation, allowing postmortem reconstruction of the implant location. In order to do so, rats were perfused transcardially with saline and 10% formalin, their brains extracted and placed in 30% sucrose for 3–5 d. Brains were then frozen; coronal sections were cut around the implant location using a sliding microtome, mounted on glass slides in DAPI Fluoromount-G medium (Southern Biotech) and a coverslip was applied. Epifluorescence microscopy was used to visualize DiI and DAPI. Figure 1 shows histologic reconstruction of electrode placement in pPC for two representative animals.

### Statistics

Statistical tests involved comparing distributions of quantities (preference, firing rate, effect size, decoding accuracy) over animals, trials, neurons, or sessions These distributions followed approximately normal distributions. Repeated measures (i.e., preference pre and post conditioning, activity during stimulus and baseline periods) were compared using a paired samples *t* test. Other comparisons were performed using an independent samples *t* test. Similarly, factors in ANOVA were treated as within-subjects or between-subjects as applicable. Frequency of occurrence was compared between conditions using nonparametric sign-test or χ^2^ test. All tests were two-tailed with α = 0.05.

## Results

### Behavioral assessment of taste-odor association learning

**Figure 2.**
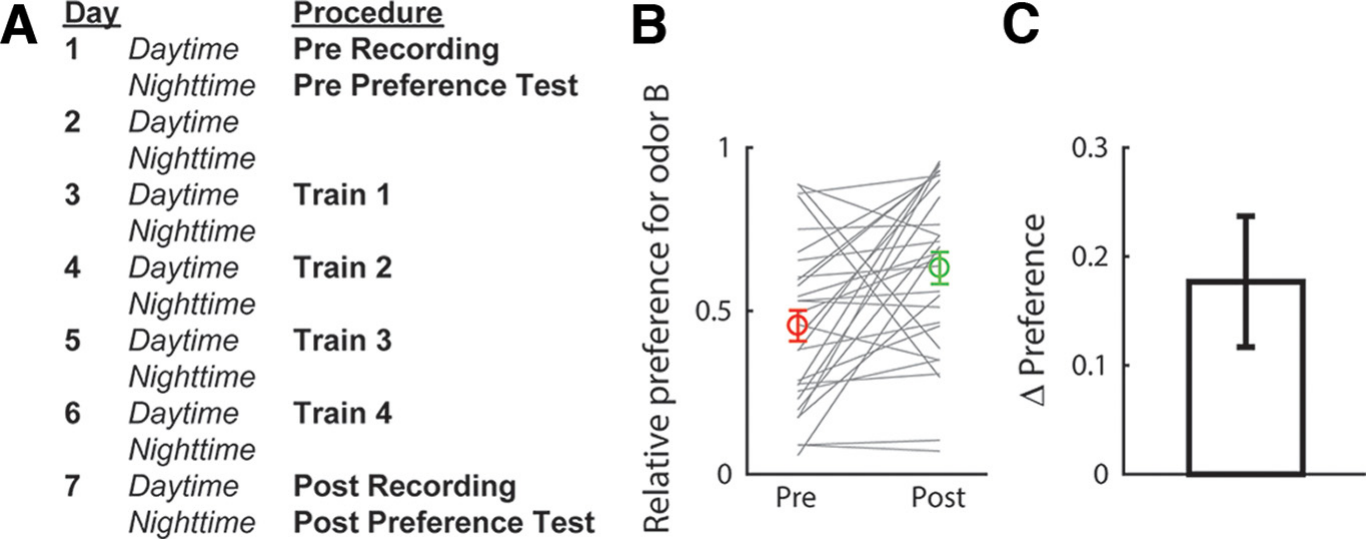
Odor-taste association paradigm. ***A***, Sequence of procedures in the experimental paradigm. ***B***, Preferences for the saccharin-paired odor (odor B) obtained before and after conditioning for each animal (gray lines). Averages (±SEM) over animals (*n* = 29) are shown in color. ***C***, Change in preference (post–pre conditioning), averaged (±SEM) over animals.

The present study aimed to elucidate the neural representation of taste-odor associations. In particular, we tested the hypothesis that taste associations of odors are encoded in the activity of pPC neurons via ongoing top-down modulations. Odor-taste association learning was established and assessed using a two-bottle consumption task in which we measured relative preference for a test odor B versus a control odor A, before and after animals learned to associate odor B with 0.2% saccharin (a palatable sweet taste). An outline of the experimental protocol is shown in Figure 2*A*. Figure 2*B* shows preferences for odor B (relative to odor A) before and after conditioning. After conditioning, a significant majority of animals (23 out of 29, 79%, sign test: *p* < 0.01) increased their preference for the saccharin-paired odor (*t* test comparing preferences before and after conditioning: *t*_(28)_ = 2.94, *p* < 0.01; Fig. 2*C*). Thus, our training procedure was effective in conditioning a preference for the saccharin-paired odor relative to the control odor, consistent with previous work (Holman, 1975; Maier et al., 2015; Blankenship et al., 2019; Christensen et al., 2022).

### Responsiveness of single pPC neurons to odor solutions

**Figure 3.**
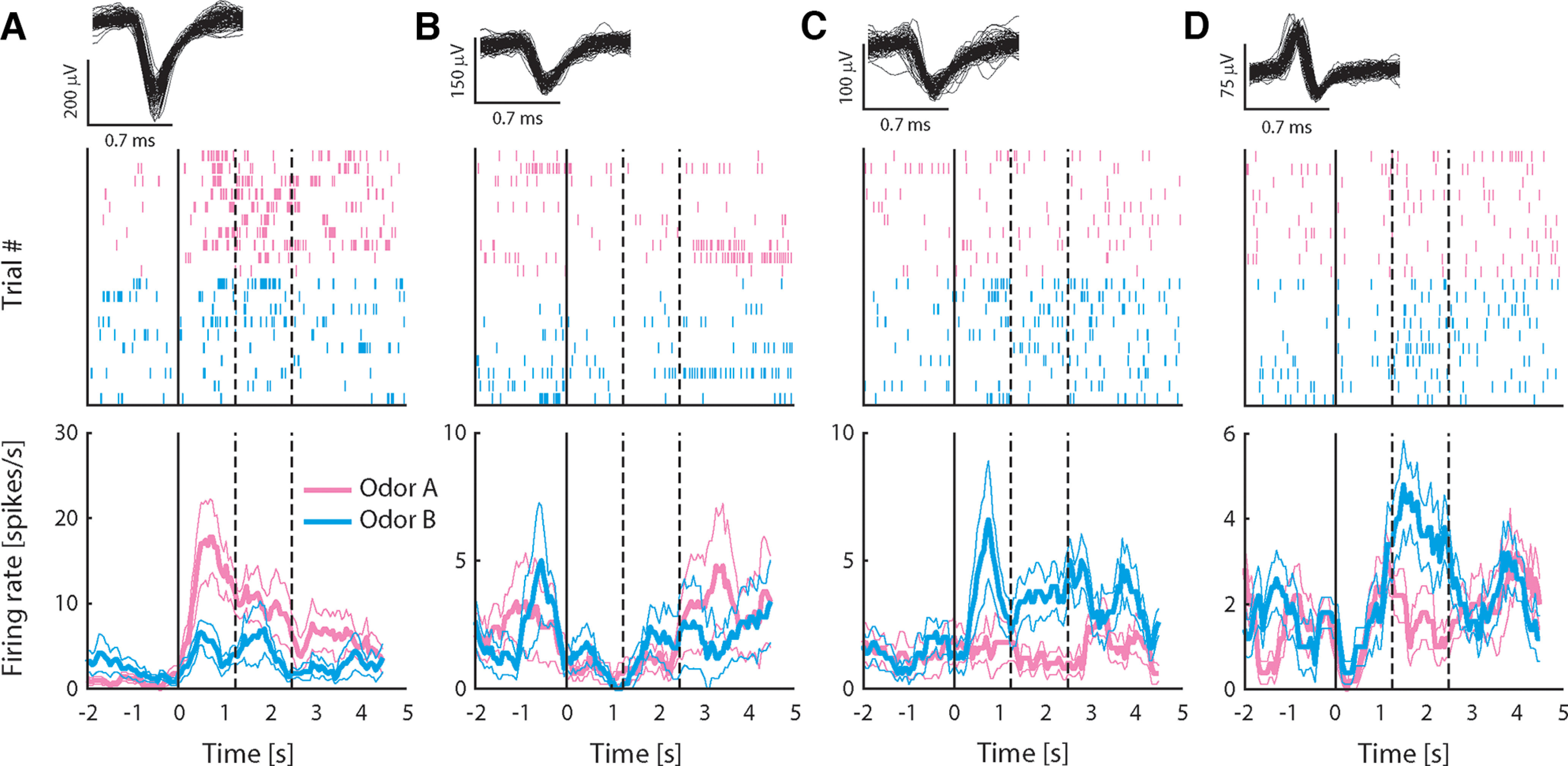
Responses to intraoral odor solutions recorded from exemplar pPC neurons. ***A–D***, Top panels show 100 randomly selected waveforms (gray) in the single neuron cluster; middle panels are spike raster plots showing all action potentials for all trials aligned on stimulus delivery (*t* = 0); and bottom panels show average firing rate (±SEM) over trials in response to odors A and B. Rate plots are calculated using a 500-ms sliding window to illustrate the temporal profile of responses, but lack the temporal resolution of raster plots because of the size of the smoothing window. Neurons in ***A***, ***B*** were recorded before conditioning; neurons in ***C***, ***D*** after conditioning. Dashed lines indicate offset of “early” and “late” response epochs.

**Figure 4.**
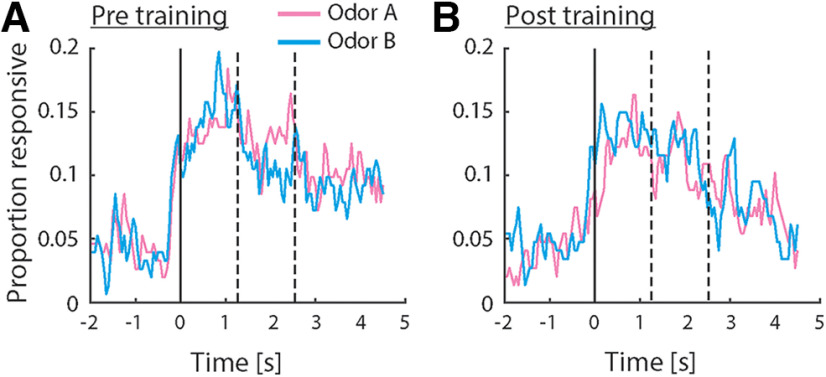
Odor responsiveness in the population of pPC neurons. ***A***, ***B***, Proportion of pPC neurons exhibiting a significant response relative to baseline, before (***A***) and after (***B***) conditioning. Responsiveness was calculated using a 500-ms sliding window. Dashed lines indicate offset of “early” and “late” response epochs. No significant differences in responsiveness were observed (χ^2^ test comparing proportions between groups).

To investigate how neurons in pPC may encode taste-odor associations, we recorded responses from small ensembles of single pPC neurons to intraoral delivery of odor A and B solutions before and after preference learning. Stimuli were presented intraorally to allow, as much as possible, the natural dynamics of sensory stimulation that occurs during oral evaluation of flavor, including retronasal odorant delivery, concurrent somatosensory stimulation and orofacial behaviors associated with consumption. A total of 299 single neurons were isolated across the two recording sessions per animal (*n* = 152 and 147 neurons before and after conditioning, respectively). Figure 3 shows examples of single pPC neuron responses, highlighting the main patterns observed across the population. The neurons in Figure 3*A*,*B* were recorded before conditioning; the neurons in Figure 3*C*,*D* were recorded after conditioning. Responses were often odor selective [Fig. 3*A*,*C*,*D*, where responses differed between odors A and B as determined by independent samples *t* test comparing average responses in stimulus period (2.5-s window immediately following stimulus onset)]; responses typically exhibited sustained responses across the stimulus period; and could be excited (Fig. 3*A*,*C*) or inhibited (Fig. 3*B*) relative to baseline [as determined by paired samples *t* test comparing average responses in the stimulus period versus baseline (2.5 s immediately preceding stimulus onset)]. Some responses appeared to exhibit complex dynamics. For example, the response to odor B for the neuron shown in Figure 3*D* switched from being inhibited early in the response to being excited later in the response. In order to determine how pPC neurons may represent information about the odor stimuli, we first quantified the number of neurons that respond to each odor. Given the variable and protracted response dynamics observed in individual neuron response profiles (see Fig. 3), we performed a sliding window analysis, comparing responses in overlapping 500 ms bins to baseline using a paired samples *t* test. Figure 4*A*,*B* shows the number of neurons that responded significantly to odors A and B as a function of time following stimulus delivery. Overall, each odor evoked sparse responses in pPC (up to 20% of total neurons at any point in time during the stimulus period), in line with previous work using orthonasal as well as retronasal odorants. The number of neurons did not differ between odors, or between pre and post conditioning (χ^2^ test: *p* > 0.05 for all time windows).

### Dynamics of odor selectivity in single pPC neurons change after conditioning

**Figure 5.**
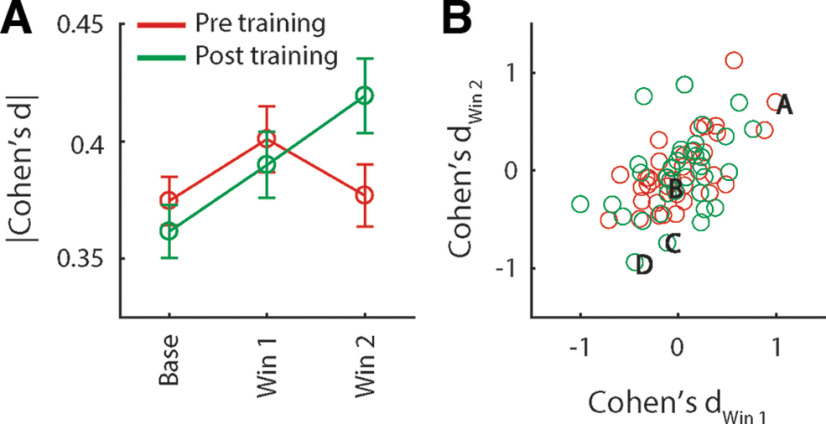
Discriminability between odors ***A***, ***B*** at the single neuron level. ***A***, Effect size (Cohen’s *d*) of the difference in the response to odors A and B, averaged (±SEM) over all neurons recorded before and after conditioning (*n* = 152 and 147, respectively), during baseline, and early and late stimulus response epochs. ***B***, Cohen’s *d* obtained from the early and late response epochs for all odor-responsive neuron (*n* = 36 before and after conditioning). Letters correspond to the example responses in Figure 3.

The example responses shown in Figure 3 demonstrate that single pPC neuron responses can respond to multiple odors in a more or less selective manner. This pattern is generally in line with previous work using orthonasal odorants showing that different odors elicit responses in partly overlapping ensembles of piriform cortex neurons. To determine how well individual neurons distinguish between odors A and B, we analyzed neurons that exhibited significant overall responsiveness, defined as a significant response to odor (pooled across odors A and B) in the stimulus period versus baseline (*n* = 36 neurons before and after conditioning). Cohen’s *d* was used as a continuous measure of discriminability. We calculated discriminability in two time windows (0–1250 and 1250–2500 ms following stimulus delivery). Figure 5*A* shows average discriminability before and after conditioning during the early and late time windows, as well as during the baseline period. Two-way ANOVA on the magnitude of discriminability (i.e., the absolute value of Cohen’s *d*) with factors Window (early, late) and Epoch (pre, post) revealed a significant Window × Epoch interaction (*F*_(1,279)_ = 4.48, *p* < 0.05). *Post hoc* comparisons (Bonferroni corrected) indicated that discriminability was significantly increased during the early portion of the response relative to baseline, and that during the late portion of the response, discriminability was significantly higher post versus pre conditioning. The increase in discriminability late in the response observed after conditioning could be the result of two scenarios. First, individual neurons could sustain their activity patterns established early in response (e.g., the neuron shown in Fig. 3*C* that prefers odor B over A during both the early and late portion of the response). Alternatively, activity patterns could differ between the early and late portion of the response (e.g., the neuron shown in Fig. 3*D* that is nonselectively early in the response, followed by an increase in firing rate in response to odor B over A later in the response). In the first scenario, raw Cohen’s *d* values obtained from the two response windows would be highly correlated. That is, the early and late portions of the response should exhibit similar odor selectivity. In the second scenario, raw Cohen’s *d* values obtained from the two response windows may be unrelated or even anticorrelated. Figure 5*B* shows raw Cohen’s *d* values obtained from the two response windows for all stimulus responsive neurons recorded pre and post conditioning. Pre conditioning, odor discriminability is highly correlated between the two response windows (*r* = 0.68, *p* < 0.001), suggesting that odor selectivity established early in the response is to some extent sustained during the late portion of the response. Post conditioning, correlation between the two response windows is significantly reduced (*F* test comparing correlations obtained pre and post conditioning: z = 1.78, *p* < 0.05), despite the fact that discriminability is significant in the late portion of the response, and is, if anything, increased relative to the early portion of the response. This pattern of results is consistent with the second scenario, in which odor-evoked responses undergo pronounced qualitative changes over the course of the response post conditioning. Thus, odor-taste conditioning is associated with changes in the pattern of responses during the late epoch, such that odor discriminability is increased.

### Odor information is present in single trial pPC ensemble responses

**Figure 6.**
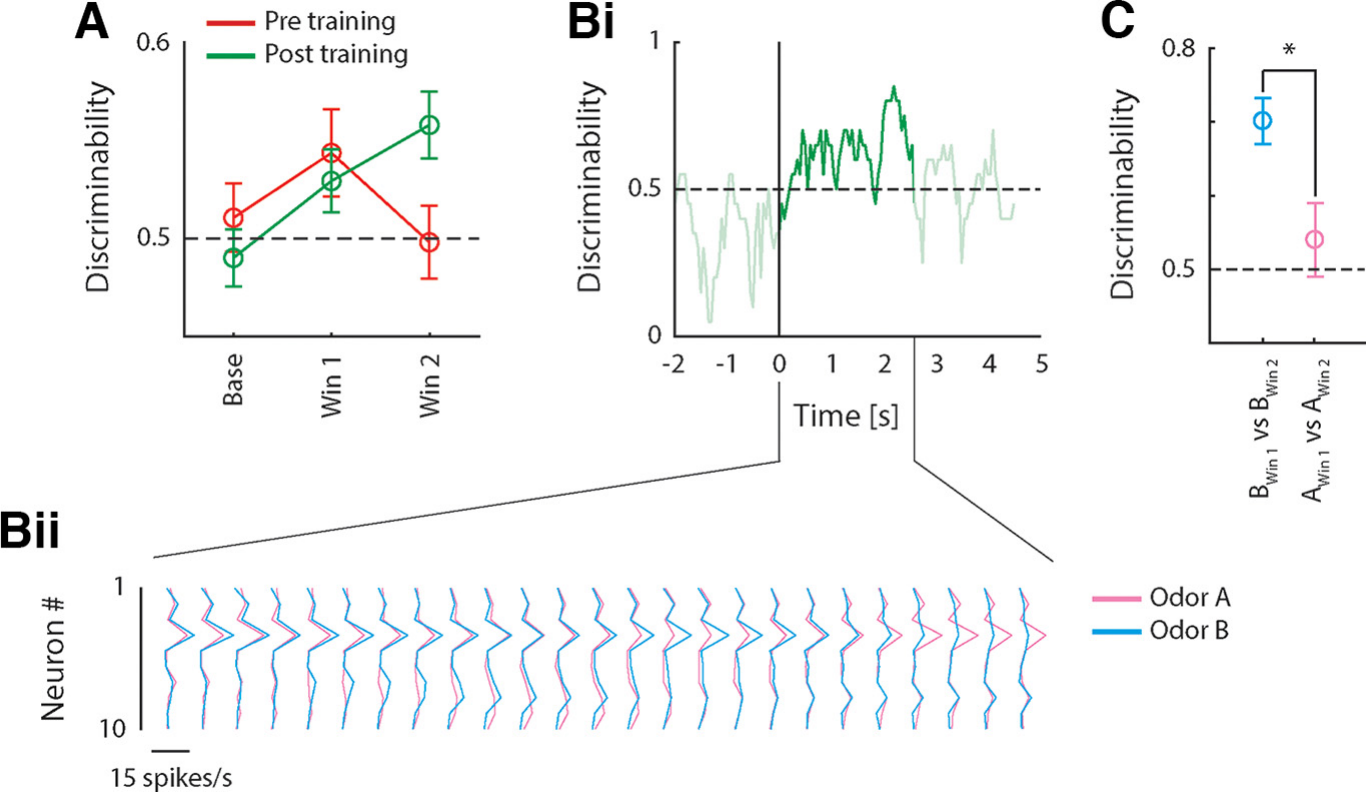
Single-trial decoding of odor identity from ensemble responses. ***A***, Discriminability between odors A and ***B***, averaged (±SEM) over all sessions with *n* > 1 neurons before and after conditioning (*n* = 20), during baseline, and early and late stimulus response epochs. ***Bi***, Discriminability as a function of time for an example ensemble recorded after conditioning (*n* = 10 neurons). Stimulus period (2500 ms following stimulus onset) is highlighted. ***Bii***, Firing rate patterns for all neurons in the example ensemble shown in ***Bi***. Responses are shown in a series of sliding windows (500-ms window size, 100-ms step size). ***C***, Discriminability of ensemble responses to the same odor in different response epochs. *t test: *p* < 0.05.

The analyses described above assessed responsiveness and discriminability of odors A and B at the level of single pPC neurons that were selected based on arbitrary response properties (i.e., significant responsiveness vs baseline). However, across the population of animals, few neurons exhibited significant responses, and the analysis presented above thus only takes into account a small sample of the total recorded neural activity. Next, we applied a more inclusive analysis, using all simultaneously recorded neural activity during individual sessions to determine whether odor identity could be decoded from the complete ensemble response on a single-trial basis. This analysis takes into account subtle variations in responses across conditions, and trials within conditions, variability that is lost in the single-neuron-based analyses presented above, and thus more closely mimics real-world conditions where animals need to identify an odor during a single encounter using the entire population of pPC responses. For each odor, we calculated an ensemble response profile consisting of a vector of firing rates (one for each simultaneously-recorded neuron, averaged across the stimulus period). Discriminability of odors A and B was calculated by comparing the distance between each single-trial ensemble response profile and each of two trial-averaged ensemble response templates (one for odor A; one for odor B). A trial was classified correctly if the distance between the trial and the matching template was smallest (i.e., when the distance between an odor A trial and the odor A template was smaller than the distance between the odor A trial and the odor B template, and vice versa). Figure 6*A* shows the average decoding performance over all sessions that featured at least two simultaneously recorded neurons [*n* = 20 sessions both pre and post conditioning; mean number of neurons per ensemble (pre/post): 6.3/6.7; range: 2–16/2–12]. Overall, the pattern of results is consistent with the one obtained from single neurons (see Fig. 5*A*): early in the response, ensembles recorded both pre and post conditioning performed above chance. However, whereas decoding performance pre conditioning dropped during the late portion of the response, performance post conditioning remained high. This pattern was confirmed by ANOVA, revealing a Window × Epoch interaction (*F*_(1,37)_ = 5.79, *p* < 0.05). Next, we examined more closely the firing rate patterns on which decoding performance is based. Figure 6*Bi* shows decoding performance obtained from an example ensemble of 10 simultaneously recorded neurons post conditioning in a sliding window over time. The dynamics of the binned ensemble response during the stimulus period is shown in Figure 6*Bii*. Although decoding performance was mostly above chance during the entire stimulus period, response patterns underwent profound changes over the course of the response, such that neurons preferring odor A early in the response switch to preferring odor B later in the response, and vice versa. Such changes in selectivity are consistent with the results from the single neuron analysis presented above (see Fig. 5*B*), suggesting that odor representations change over the course of the response. To directly test whether this is true at the ensemble level, we compared single-trial ensemble responses to the same odor between two time windows (one early in the response; one late in the response). In other words, we asked the question: How well can we decode odor identity from late epoch ensemble responses using templates obtained from the early epoch? Figure 6*C* shows the result of this analysis. Ensemble responses to odor B during early and late epochs were easily distinguished, indicating that responses to odor B differed between epochs. In contrast, ensemble responses to odor A were more similar between epochs (*t* test comparing discriminability of early and late epoch responses between odors A and B: *t*_(12)_ = 2.46, *p* < 0.05). No differences were found in between-epoch discriminability of responses to the same odors pre conditioning, a pattern that differed significantly from the one observed post conditioning (*t* test comparing the difference in discriminability between odors A and B pre versus post conditioning: *t*_(19)_ = 2.60, *p* < 0.05). Together, results from the decoding analysis demonstrate that odor identity can be read out early in the response using relatively small ensembles of pPC neurons, regardless of conditioning. Conditioning further enhances discriminability between conditioned and unconditioned odors in a manner that is selective to the late response epoch, and is characterized by a unique ensemble response code.

### Ensemble decoding accuracy does not depend on palatability

**Figure 7.**
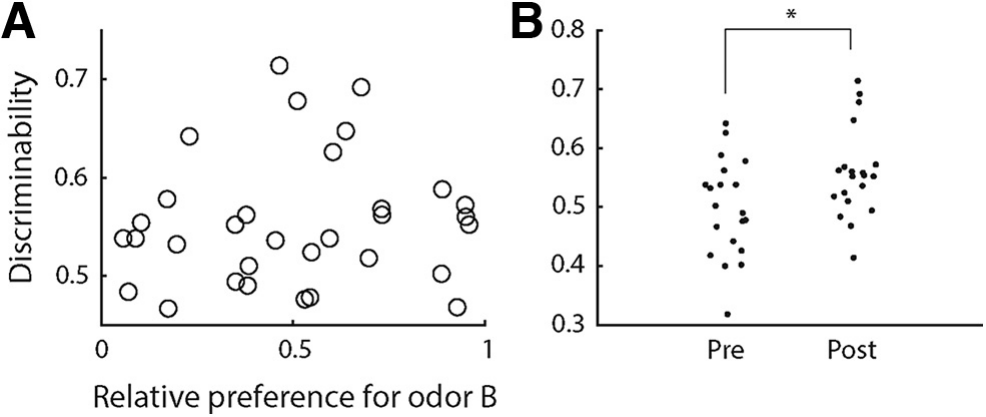
Relation between ensemble decoding accuracy and odor palatability. ***A***, Ensemble discriminability between odors A and B during the late response epoch as a function of relative preference for odor B for each animal (*n* = 19). No significant relation was detected by linear regression. ***B***, Ensemble discriminability between odors A and B during the late response epoch before and after conditioning for each animal (*n* = 19). *Regression: *p* < 0.05.

The results presented above indicate that taste-odor association learning increases discriminability between the representations of odors A and B in the late portion of pPC ensemble responses. However, it is unclear what aspect of odors is reflected exactly in the response of pPC neurons. One possibility is that the late response epoch reflects odor palatability. Our behavioral results (Fig. 2) show that the palatability of the saccharin-associated odor increases after conditioning, and this increase in palatability could therefore be driving changes in pPC ensemble response patterns. To directly test for this possibility, we performed linear regression of ensemble discriminability during the late epoch on preference for odor B (Fig. 7*A*). This analysis yielded no significant effect of preference on odor discriminability (*F* = 1.46, *p* = 0.24). Regression using pre versus post conditioning as a predictor (Fig. 7*B*) confirmed that discriminability increased following association learning (*F* = 5.70, *p* < 0.05). Thus, these findings suggest that ensemble discriminability cannot be explained by odor palatability, but instead reflects other aspects of odor perception.

## Discussion

Our results demonstrate that taste-odor association learning changes responses of pPC neurons to odor solutions. Association-related changes in responsiveness appeared as changes in the dynamics of stimulus-evoked responses, leading to enhanced discriminability between representations of taste-associated and nontaste-associated odors. Learning-related activity patterns were selective to the late portion of the response, and qualitatively different from the patterns observed during the early portion of the response. We speculate that during oral evaluation of odor stimuli, association-related representations are generated by ongoing interactions with the taste system and can be read-out by downstream brain areas independently of chemical identity representations.

Experience-dependent coding of olfactory information is supported by the organization of the primary olfactory (piriform) cortex. Information about odorant identity is relayed from olfactory sensory neurons to the piriform cortex via the olfactory bulb, and is represented in unique but partly overlapping ensembles of neurons that are distributed throughout the piriform cortex without apparent spatial organization (Scott et al., 1980; Schwob and Price, 1984; Rennaker et al., 2007; Stettler and Axel, 2009; Miyamichi et al., 2011; Sosulski et al., 2011). Besides bottom-up sensory input from the olfactory bulb, piriform cortex also receives inputs from various extraolfactory systems (Luskin and Price, 1983; Johnson et al., 2000; Haberly, 2001; Majak et al., 2004; Sadrian and Wilson, 2015). Our findings are generally in line with previous work demonstrating that experience can shape cortical odor representations (Kadohisa and Wilson, 2006; W. Li et al., 2006, 2008; Calu et al., 2007; Roesch et al., 2007; Barnes et al., 2011; Chen et al., 2011; Chapuis and Wilson, 2012; Gire et al., 2013; Meissner-Bernard et al., 2019; D. Wang et al., 2019), and shed new light on the mechanisms underlying context-dependent cortical olfactory coding. One possible way of encoding experience is through plasticity in the local cortical circuit. In this scenario, experience affects how bottom-up inputs from the bulb are processed by the local cortical circuit, resulting in a static rearrangement of odor relationships. For example, Chapuis and colleagues (Chapuis and Wilson, 2012) showed that cortical representations of the components of an odor mixture may become more or less similar to each other, depending on whether rats were rewarded to respond to the mixture in a configural or elemental manner, respectively. In addition to experience-dependent changes in the local representation of bottom-up input patterns, some studies have suggested that piriform cortex may explicitly encode associative features of odor stimuli independently of chemical identity (Calu et al., 2007; Roesch et al., 2007; Gire et al., 2013; Meissner-Bernard et al., 2019; D. Wang et al., 2019). Conversely, others have suggested that explicit encoding of associative features occurs elsewhere in the brain (Millman and Murthy, 2020; P.Y. Wang et al., 2020).

The present findings suggest that pPC neurons explicitly encode taste associations of odors in the temporal dynamics of their response, uncovering new insight into the mechanisms underlying context-dependent cortical odor coding. Experience-dependent changes did not appear as static changes in odor representations. That is, changes were not uniform across the response period. Instead, experience-dependent changes were confined to the late portion of the response. Changes in response dynamics following conditioning did not simply sustain the representation observed during the early portion of the response, but led to the emergence of a new representation. Taken together, these findings suggests that pPC neurons multiplex information using a dynamic coding scheme, where different aspects of an odor stimulus are represented during different epochs over the course of the response. With respect to the early response period (initial 1 s following stimulus delivery), we speculate, in line with previous work on piriform cortical odor coding, that activity patterns reflect bottom-up input from the olfactory bulb containing information about odor identity (Rennaker et al., 2007; Poo and Isaacson, 2009; Stettler and Axel, 2009; Bolding and Franks, 2017). With respect odor information that appeared selectively after conditioning during the late response period (after 1 s following stimulus deliver), activity patterns may encode taste associations of odors; alternatively, they may reflect hedonic value of odors. The former interpretation is consistent with the proposed role for piriform cortex in stimulus identity processing, and pPC neurons may dynamically represent chemical odor identity and associated taste identity. Indeed, behavioral work in humans suggests that taste associations of odors can be taste specific (Stevenson et al., 1995, 1998, 2000a, b; Stevenson and Boakes, 2004; Yeomans et al., 2006). Moreover, we did not find any evidence that the late portion of the pPC response reflects odor palatability. Future work in which animals learn to associate different odors with different tastes that vary in quality and hedonic value will more explicitly characterize the nature of information encoded in the different response epochs.

Few studies to date have explicitly considered the temporal dynamics of cortical odor responses and their relation to associative coding. One notable exception is a study by Gire and colleagues (Gire et al., 2013), who recorded from neurons in the posterior piriform cortex of rats to rewarded and unrewarded odors. They considered the contribution of activity patterns at two temporal scales: inhalation-locked and noninhalation-locked. Whereas information about chemical identity was present in fast, inhalation-locked responses that are thought to reflect processing of bottom-up inputs by local piriform cortical circuits (Bolding and Franks, 2018), information about reward value was present in slower activity patterns that were not locked to respiration. The slow dynamics of experience-dependent activity pattern observed in the present study and by Gire et al. (2013) suggests that they are conveyed to pPC by top-down inputs. Together, findings from both studies support a model in which associative odor coding emerges dynamically through ongoing interactions with extraolfactory systems. More broadly, a dynamic model of odor coding in pPC is in line with previous observations of response dynamics at time scales beyond the sniff cycle (Rennaker et al., 2007; D. Wang et al., 2019).

Dynamic multiplexing of sensory and associative information at timescales similar to the ones observed in the present study has previously been observed in other brain regions that process consumption-related sensory cues. For example, activity patterns in the insular gustatory cortex reflect taste identity during the initial second following intraoral taste delivery; followed by activity patterns that reflect hedonic value of the taste stimulus, independent of stimulus identity (Katz et al., 2001; Sadacca et al., 2012). Moreover, EMG recordings of orofacial movements in response to intraoral taste solutions demonstrate that changes in GC neuron response patterns precede the expression of palatability-related behavioral responses, suggesting a causal role for response dynamics in controlling behavior (J.X. Li et al., 2016; Mukherjee et al., 2019). These response dynamics arise from ongoing interactions with the basolateral amygdala (Fontanini et al., 2009; Piette et al., 2012; Lin et al., 2021). In the present study, we did not measure behavioral responses on a trial-by-trial basis, but the temporal scale of the observed dynamics is broadly consistent with a role in oral evaluation of sensory stimuli. Future work measuring orofacial movements (e.g., mouth movements, sniffing) in real time will determine the temporal relation between behavioral and neural response patterns.

The effects of conditioning observed in the present study constitute relatively subtle changes in neural response patterns. Odor responsiveness in individual neurons was overall sparse, and conditioning did not result in significant changes in responsiveness of individual neurons (i.e., we did not observe an increase or decrease in the number of neurons that responded significantly to odor stimuli). The lack of sensitivity in single neuron analysis may be because of a high degree of response variability. One factor that likely contributed to response variability across trials is the protracted nature of the response. Responses of single pPC neurons unfolded over seconds following stimulus delivery and are poorly locked to stimulus onset. Another source of variability is that neural activity appeared to change in a heterogenous manner, with some neurons increasing and others decreasing their activity levels in a dynamic manner. These issues underscore the value of ensemble analysis that takes into account correlated, heterogeneous changes in neural activity across multiple neurons on a single trial basis. Indeed, our ensemble decoding analysis was able to significantly discriminate between odor representations using responses from relatively small ensembles of pPC neurons.

The changes in pPC responsiveness following taste association learning observed here are reminiscent of the changes in pPC responses to odor solutions when presented in mixture with taste compounds. We recently demonstrated that taste and odor components of mixtures interact to change responses of pPC neurons in real time (Idris et al., 2023). Multisensory modulations observed in that study resembled the experience-dependent modulations observed here in that they were mostly subtle at the single neuron level, heterogenous in nature, and led to greater discriminability of odor representations at the ensemble level. Also in line with the present findings, increased discriminability resulting from real-time multisensory modulation was not observed until later in the response, following an initial unisensory odor-selective phase. The fact that we recorded from separate populations of neurons before and after conditioning precludes a direct comparisons between real-time and experience-driven pPC response modulation, future work aimed at tracking the activity of single neurons across conditioning will determine whether real-time interactions between taste and smell components of mixture are predictive of experience-dependent modulations.

Regarding potential extraolfactory sources relaying associative input about taste, previous work has identified GC as a candidate region, as inactivation of GC prohibited the expression of preferences for sweet taste-associated odors (Maier et al., 2015; Blankenship et al., 2019). Based on previous findings, the influence of GC on olfactory processing appears to be specific to the context of consumption. Intraoral delivery of odors in solution creates a unique context that has been shown to play a key role in mediating taste-odor association learning. Blankenship et al. (2019) demonstrated that animals more readily learn preferences for sweet taste-associated odor when odors were presented intraorally versus orthonasally, and that inactivating GC selectively affected the expression of preferences for intraorally presented sweet taste-associated odors. Further evidence for unique processing of consumption-related odor signals comes from human imaging work, showing increased BOLD signal in GC in response to food odors versus nonfood odors (Veldhuizen et al., 2010). Although it remains unclear whether the response patterns observed in the present study are unique to the context of consumption, pPC neurons have been shown to be sensitive to oral context: responses to the same odorant presented intraorally and orthonasally can differ substantially (Maier, 2017). Together, these findings suggest that oral context may play a key role in generating the response patterns observed in the present study.

## Synthesis

Reviewing Editor: Arianna Maffei, Stony Brook University Decisions are customarily a result of the Reviewing Editor and the peer reviewers coming together and discussing their recommendations until a consensus is reached. When revisions are invited, a fact-based synthesis statement explaining their decision and outlining what is needed to prepare a revision will be listed below. The following reviewer(s) agreed to reveal their identity: Donald Wilson.

The reviewers agree that this is an interesting study that provides a novel contribution to the field.

They made recommendations to strengthen the manuscript and clarify some issues. One important point regards the use of data obtained from animals that were used for other experiments included in a published study. This should be noted in the methods and possible issues that may arise for having used the same animals for multiple studies should be addressed. The degree of overlap in data collection and figures should also be addressed.

The reviewers also identified other points in the manuscript that would benefit from clarifications.

The full set of comments by the reviewers follows below.

Reviewer #1

This manuscript provides a well-written investigation into how neurons in the piriform cortex dynamically represent gustatory-odor sensory associations before and after learning odor-tastant pairs, a feat not yet accomplished within the field. This builds off of prior work implicating the piriform cortex as a region of interest in processing odor-taste associations, and provides unique insight into how these flavor preferences are learned. We appreciated the well-executed behavioral paradigm coupled with careful ensemble analyses to investigate how experience shapes neural responses to olfactory information and taste integration. This is a nice paper and an advance for the field.

comments:

Apologize if we are incorrectly concluding: this study appears to be a presentation of data acquired from the same rats (?, and likely same units?) published in a prior publication (PMCID: PMC9869978) by this group. Perhaps due to the authors desire to provide a double-blind submission, which we appreciate, there is no clear mention that this study includes data / animals also published in the groups recent JPhysiology paper (PMCID above). It is obvious at least some of the same rats and recordings were used since even Figure 1 in this paper, which shows electrode histology, seems EXACTLY the same as what the group published in the prior paper (which would be a copyright issue by the way). some solutions to suggest for improving this present paper largely center around the need to be more forthcoming regarding the relationship between the prior publication and this one:

The authors should include variants of text in the introduction and methods both indicating that these animals were used for this other study, as was done in the Results section of PMCID: PMC9869978: “However, animals were part of a variety of larger studies, and continued into additional recordings and behavioural tests following the recording of naïve responses reported here.”

The authors should address the potential implications for how these other experiments could had impacted the rats’ behavior and neural responses should be discussed. Was there any chance for a testing/recording order effect for instance?

We would also appreciate for the authors to include a new paragraph or more in the discussion comparing and contrasting this present manuscript with the prior publication. And specifically to compare/contrast if the response of the cells in the present manuscript with those of the previously published paper to further characterize how these cells respond differently over the course of learning. What is similar in terms of behavior and coding in this paper to the prior paper? Same %’s and/or numbers of cells showing odor responses, taste responses, integrating both, etc? any additional comments/discussions along these lines the authors think they may be able to include are welcome as well. by the end of the discussion, the readers should be left with a clear picture for what is new, what was shared, and among that shared, how replicated the results were across analyses in the two publications.

Of minor note, in the figure caption for Figure 4, starting in line 521, the authors state “Dashed lines indicate offset of ‘early’ and ‘late’ response epochs,” however there are no dashed lines in the actual figure. To maintain consistency with Figure 3, the addition of those dashed lines would be helpful.

Reviewer #2

This manuscript further strengthens the basis for considering the posterior piriform cortex as an associative, multisensory cortex involved in chemosensory memory. The results demonstrate associative experience (taste-odor association) modifies pPC dynamic response to odor, with early responses largely reflecting odor quality and later response (>1sec) reflecting aspects of the learned taste association. The experimental design and analyses appear strong - though see note below and the interpretation well integrated with the literature. In fact, although a bit long, the Discussion is outstanding in discussing the results and their implications without drifting into speculation. The discussion of bottom-up and top-down processes potentially involved is important in understanding this structure and clearly suggests exciting follow-up work.

Major

Line 130 - “Unisensory odor stimuli were exemplars of monomolecular odorants”. Interpretation of the work depends on the odorants not having gustatory or trigeminal activity, as this sentence implies. What is the evidence that methyl valerate and 2-hexanone are unisensory?

Very minor

Line 84 - “that that”

Line 102 - “with the XXXX animal care committee’s”

Line 118 - “or multi-electrodes silicon probes” should be ‘electrode’

## Author Response

Synthesis of Reviews:

Synthesis Statement for Author (Required):

The reviewers agree that this is an interesting study that provides a novel contribution to the field.

They made recommendations to strengthen the manuscript and clarify some issues. One important point regards the use of data obtained from animals that were used for other experiments included in a published study. This should be noted in the methods and possible issues that may arise for having used the same animals for multiple studies should be addressed. The degree of overlap in data collection and figures should also be addressed.

The reviewers also identified other points in the manuscript that would benefit from clarifications.

The full set of comments by the reviewers follows below.

Reviewer #1

This manuscript provides a well-written investigation into how neurons in the piriform cortex dynamically represent gustatory-odor sensory associations before and after learning odor-tastant pairs, a feat not yet accomplished within the field. This builds off of prior work implicating the piriform cortex as a region of interest in processing odor-taste associations, and provides unique insight into how these flavor preferences are learned. We appreciated the well-executed behavioral paradigm coupled with careful ensemble analyses to investigate how experience shapes neural responses to olfactory information and taste integration. This is a nice paper and an advance for the field. comments:

Apologize if we are incorrectly concluding: this study appears to be a presentation of data acquired from the same rats (?, and likely same units?) published in a prior publication (PMCID: PMC9869978) by this group. Perhaps due to the authors desire to provide a double-blind submission, which we appreciate, there is no clear mention that this study includes data / animals also published in the groups recent JPhysiology paper (PMCID above). It is obvious at least some of the same rats and recordings were used since even Figure 1 in this paper, which shows electrode histology, seems EXACTLY the same as what the group published in the prior paper (which would be a copyright issue by the way). some solutions to suggest for improving this present paper largely center around the need to be more forthcoming regarding the relationship between the prior publication and this one: 2

Thank you for bringing up the potential copyright issue. I have modified the Figure slightly, but I leave it to the editor/journal administration to make a recommendation on how to best deal with this. The authors should include variants of text in the introduction and methods both indicating that these

animals were used for this other study, as was done in the Results section of PMCID: PMC9869978:

“However, animals were part of a variety of larger studies, and continued into additional recordings and behavioural tests following the recording of naïve responses reported here.”

We fully agree with the reviewer. We have added the explicit reference to the Methods section (L173- 180).

The authors should address the potential implications for how these other experiments could had impacted the rats’ behavior and neural responses should be discussed. Was there any chance for a testing/recording order effect for instance?

We now describe the exact nature of the exposure and address its potential effects on the present results in the Methods section (L173-180).

We would also appreciate for the authors to include a new paragraph or more in the discussion comparing and contrasting this present manuscript with the prior publication. And specifically to compare/contrast if the response of the cells in the present manuscript with those of the previously published paper to further characterize how these cells respond differently over the course of learning.

What is similar in terms of behavior and coding in this paper to the prior paper? Same %’s and/or numbers of cells showing odor responses, taste responses, integrating both, etc? any additional comments/discussions along these lines the authors think they may be able to include are welcome as well. by the end of the discussion, the readers should be left with a clear picture for what is new, what was shared, and among that shared, how replicated the results were across analyses in the two publications.

We agree that this makes for an interesting comparison. We have added a paragraph to the discussion comparing the findings from both papers and how response patterns reported in both papers may relate to each other. Note that explicit quantitative comparison of response modulations is made less meaningful by the current design, and will instead be the topic of future work (L486-499). Of minor note, in the figure caption for Figure 4, starting in line 521, the authors state “Dashed lines indicate offset of ‘early’ and ‘late’ response epochs,” however there are no dashed lines in the actual figure. To maintain consistency with Figure 3, the addition of those dashed lines would be helpful. We thank the reviewer for catching this. We have added reference lines to the Figure.

Reviewer #2

This manuscript further strengthens the basis for considering the posterior piriform cortex as an associative, multisensory cortex involved in chemosensory memory. The results demonstrate associative experience (taste-odor association) modifies pPC dynamic response to odor, with early responses largely reflecting odor quality and later response (>1sec) reflecting aspects of the learned taste association. The experimental design and analyses appear strong - though see note below and the interpretation well integrated with the literature. In fact, although a bit long, the Discussion is outstanding in discussing the results and their implications without drifting into speculation. The discussion of bottom-up and top- 3 down processes potentially involved is important in understanding this structure and clearly suggests exciting follow-up work. Major Line 130 - “Unisensory odor stimuli were exemplars of monomolecular odorants”. Interpretation of the work depends on the odorants not having gustatory or trigeminal activity, as this sentence implies. What is the evidence that methyl valerate and 2-hexanone are unisensory? We agree that this is an important consideration. There is to date no empirical evidence that the odorants used here completely lack gustatory and/or trigeminal qualities. However, we argue that both behavioral and neural responses reported here are driven by the olfactory component of the stimuli. Regarding behavioral responses: 1) We specifically selected odor concentrations that were determined in prior work to be below non-olfactory behavioral detection thresholds (Slotnick et al., 1997; Gautam & Verhagen, 2012); (2) It is highly unlikely that the increase in preference observed in our paradigm reflects a conditioned preference for the (near-threshold at best) taste of the odor solution. No previous work has demonstrated that saccharin can change the innate palatability of other tastes in mixture, whereas saccharin has repeatedly been shown to readily condition a preference for concurrently presented orthonasal odorants (Blankenship et al., 2019; Torquet et al., 2014). Regarding neural responses: 1) We previously showed that pPC responses to intra-oral odor and taste solutions differ in latency, with responses to (well-above threshold) taste solutions having a longer latency than responses to odor solutions (Maier, 2017). Such a pattern is incompatible with the explanation that responses to odor solutions are driven by (near-threshold at best) taste qualities of odor solutions; 2) For the reasons provided above with respect to behavioral responses, it is highly unlikely that changes in response patterns reflect changes in how taste components of odor solutions are perceived. Finally, with respect to potential differences in mouthfeel, virtually all odorants have trigeminal qualities. This presents a confound for all olfactory systems research (whether odorants are presented orthonasally, retronasally or intra-orally in solution). However, at the concentration used here, non-olfactory components of odor solutions are below threshold for detection (see above). We (and the rest of the field) therefore consider it highly unlikely that these potential (near threshold at best) taste qualities outweigh the well above threshold of the olfactory stimulus qualities.

We have added a summary of this response to the Methods section, and refer explicitly to previous studies (L128-138).

Very minor

Line 84 - “that that”

Line 102 - “with the XXXX animal care committee’s”

Line 118 - “or multi-electrodes silicon probes” should be ‘electrode’

We thank the reviewer for catching these typos. We corrected them in the revised manuscript. Note that the text replaced by “XXXX” contains identifiable information and in a (failed) attempt to keep the author from being identified is available in the version provided to the editor only.
